# A multicenter prediction model for false-positive clinical nodal disease in < 4-cm NSCLC: clinical implications for upfront surgery versus neoadjuvant therapy

**DOI:** 10.1186/s12885-026-16580-4

**Published:** 2026-07-17

**Authors:** Tetsuya Isaka, Yui Kaburaki, Ikki Takada, Ryotaro Matsuyama, Chiaki Kanno, Takuya Nagashima, Yujin Kudo, Yoshihiro Miyata, Morihito Okada, Norihiko Ikeda, Hiroyuki Ito

**Affiliations:** 1 Department of Thoracic Surgery, Kanagawa Cancer Center, 2-3-2 Nakao, Asahi, Yokohama, Kanagawa, 241-8515 Japan; 2 Department of Surgery, Tokyo Medical University, Tokyo, Japan; 3 Department of Surgical Oncology, Hiroshima University, Hiroshima, Japan

**Keywords:** Clinical nodal involvement, False-positive cN staging, Perioperative therapy, Positron emission tomography, Standardized uptake value

## Abstract

**Background:**

Perioperative immunochemotherapy is recommended for resectable non-small cell lung cancer (NSCLC) with ≥ 4-cm tumors or clinical nodal involvement (cN(+)). Therefore, accurate preoperative nodal assessment is crucial in < 4-cm tumors, where false-positive cN staging should be carefully considered. This multicenter study aimed to develop and validate a prediction model to identify patients at high risk of cN(+)pN(−) disease.

**Methods:**

The development cohort included 251 patients with tumors < 4 cm who were diagnosed as cN(+) using positron emission tomography and underwent curative anatomical resection with mediastinal lymph node dissection between 2010 and 2020. The model predicting cN(+)pN(−) was developed and subsequently validated in a temporally independent validation cohort of 108 patients treated during periods different from those of the development cohort.

**Results:**

In the development cohort, 72 patients (28.7%) were cN(+)pN(−). In multivariable analysis, age ≥ 67 years (odds ratio [OR] 2.85, *p* = 0.004), right-sided tumor (OR 2.50, *p* = 0.006), carcinoembryonic antigen ≤ 12 ng/mL (OR 3.11, *p* = 0.048), cN1 (OR 2.37, *p* = 0.025), and tumor maximum standardized uptake value ≤ 6.5 (OR 2.03, *p* = 0.023) were independent predictors for cN(+)pN(−). The prediction model showed a C-index of 0.74 (95% confidence interval [CI] 0.67–0.81), specificity of 94.4%, and positive predictive value (PPV) of 66.7% in the development cohort. In the validation cohort, the C-index was 0.77 (95% CI 0.67–0.86) with good calibration (slope, 1.002; intercept, 0.001), specificity (96.3%), and PPV (70.0%).

**Conclusions:**

In patients with < 4-cm cN(+) NSCLC, a simple model based on five readily available preoperative factors can identify individuals at high risk of false-positive nodal staging with high specificity. This model may help identify patients in whom upfront surgery for definitive pathological nodal evaluation may be preferable rather than immediate neoadjuvant therapy.

**Supplementary Information:**

The online version contains supplementary material available at https://doi.org/10.1186/s12885-026-16580-4.

## Background

Primary lung cancer remains a leading cause of cancer-related mortality worldwide [1]. Even in resectable non-small cell lung cancer (NSCLC), the outcomes remain suboptimal. According to the 8th edition of the TNM classification, the reported 5-year overall survival (OS) rates for clinical stages IIA, IIB, and IIIA are 15%–60%, 17%–53%, and 14%–36%, respectively [2].

Recently, the CheckMate 816 [3], KEYNOTE-671 [4], and AEGEAN trials [5] have demonstrated the efficacy of neoadjuvant or perioperative chemo-immunotherapy in clinical stage II–IIIA NSCLC without epidermal growth factor receptor (EGFR) mutations or anaplastic lymphoma kinase (ALK) fusions. Reflecting these data, the National Comprehensive Cancer Network (NCCN) guidelines [6], Japanese Lung Cancer Society guidelines [7], and National Institute for Health and Care Excellence guidance [8] recommend immunotherapy-based perioperative strategies for resectable stage II or higher disease—specifically ≥ 4-cm tumors or < 4-cm tumors with clinical nodal involvement (cN(+)) in EGFR/ALK-negative or unknown cases.

In this context, accurate preoperative nodal assessment is particularly important in < 4-cm tumors. According to the European Society of Thoracic Surgeons (ESTS), American College of Chest Physicians (ACCP), and Japanese Lung Cancer Society guidelines, endobronchial ultrasound (EBUS)/endoscopic ultrasound (EUS)-guided sampling and/or mediastinoscopy is recommended not only for radiologically suspected mediastinal disease but also in high-risk patients, including those with central tumors, cN1 disease, or tumor size > 3 cm, even when mediastinal findings are absent on imaging [7, 9, 10]. However, accurate preoperative nodal staging remains challenging because EBUS-guided transbronchial needle aspiration (EBUS-TBNA) cannot access all nodal stations [11–13] and mediastinoscopy has similar anatomical limitations that preclude access to all mediastinal nodal stations, aside from requiring general anesthesia and carrying procedural risks [14, 15]. Consequently, concordance between clinical and pathological staging remains suboptimal, with a reported clinical TNM–pathologic TNM (cTNM–pTNM) agreement of only 54.6% in a large cohort despite guideline-based staging [16] and clinical–pathological N discordance rates of 16.9%–38% in other studies [14, 17, 18].

Although neoadjuvant chemo-immunotherapy is increasingly being standardized, perioperative trials have reported grade ≥ 3 adverse events in 32.5%–44.9% of patients, grade 5 events in 0.9%–1.7% of patients, and failure to proceed to surgery in 17.9%–22.3% of patients [3–5, 19]. Moreover, according to subgroup analyses from neoadjuvant and perioperative trials, the treatment effect is more pronounced in patients with more advanced stage disease, particularly those with nodal involvement, supporting the biological rationale that preoperative systemic therapy is particularly beneficial in patients with true pathological nodal metastasis or nodal involvement [3–5]. Therefore, in < 4-cm NSCLC, cN(+) should ideally be reserved for patients with a high probability of true pathological nodal involvement (pN+). Meanwhile, prioritizing surgical resection with definitive pathological nodal evaluation may represent a reasonable alternative strategy in patients at high risk of cN(+)pN(−) disease.

Accordingly, this study aimed to identify the predictive factors for cN(+)pN(−) disease in < 4-cm NSCLC using a multicenter database, develop a high-specificity prediction model for false-positive cN staging, and validate the model in an independent cohort in order to facilitate more appropriate treatment selection.

## Methods

### Ethics statement

This retrospective multicenter database study was approved by the institutional review boards of Kanagawa Cancer Center (24EKI54, approved June 14, 2021; 2025EKI-121, approved November 25, 2025), Tokyo Medical University Hospital (SH2969), and Hiroshima University Hospital (E-1216). The requirement for individual written informed consent was waived.

### Patients

**Fig. 1:**
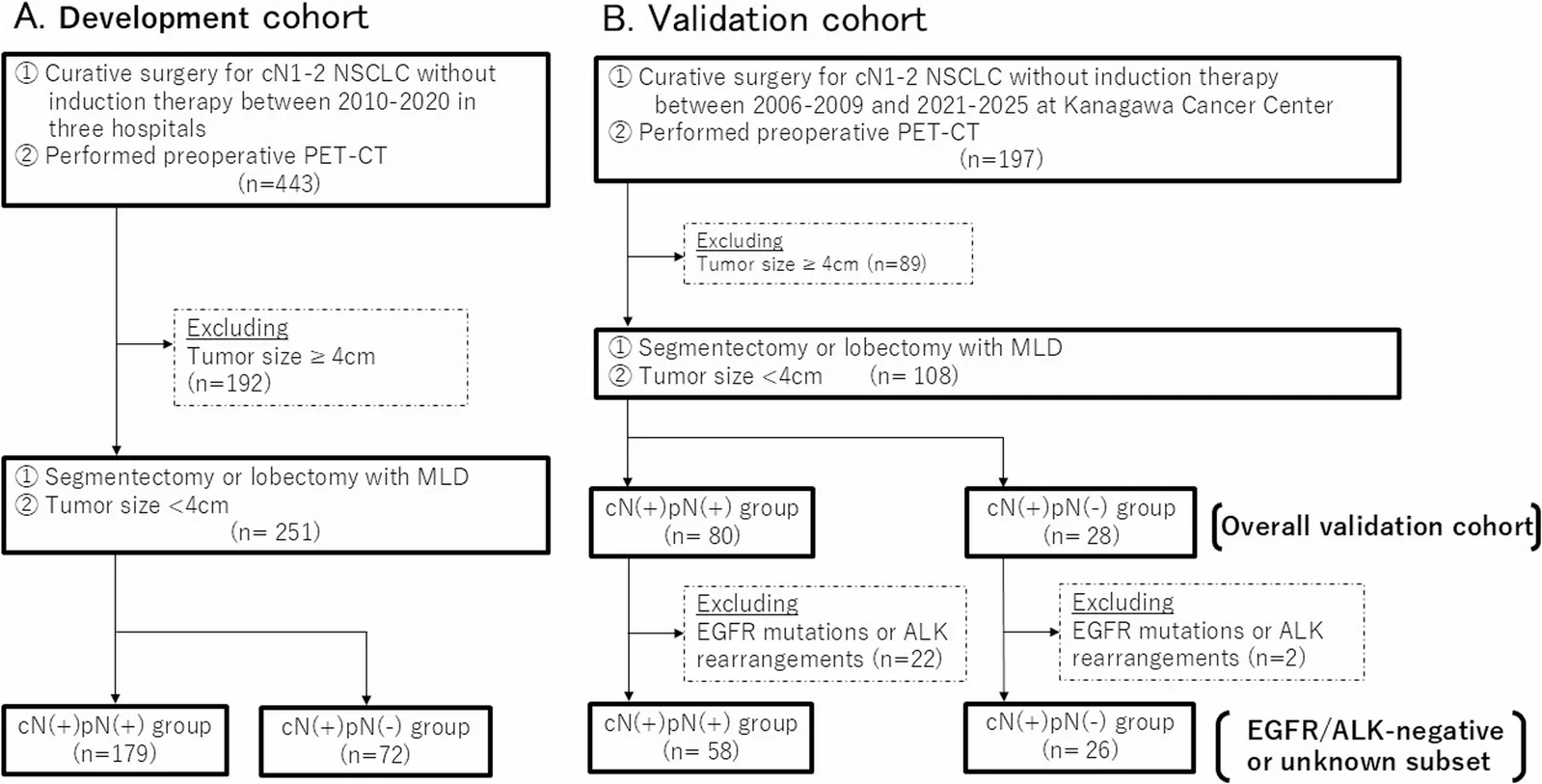
CONSORT diagram of the development and validation cohorts

The development cohort included patients who were diagnosed as cN(+) and underwent anatomical lung resection (segmentectomy or more extensive procedure) with mediastinal lymph node dissection at Kanagawa Cancer Center, Hiroshima University Hospital, or Tokyo Medical University Hospital between January 2010 and December 2020. Before surgery, all patients were treatment-naïve and underwent preoperative fluorodeoxyglucose positron emission tomography/computed tomography (FDG-PET/CT). Among the 443 eligible patients, 251 patients with tumor size < 4 cm on preoperative CT were included in the main analysis (Fig. 1a).

For temporal validation, we identified patients with NSCLC at Kanagawa Cancer Center who underwent preoperative FDG-PET/CT, were diagnosed with cN(+), and underwent anatomical resection plus mediastinal lymph node dissection during two periods: January 2006–December 2009 and January 2021–December 2025. Patients who were treated during the development period (2010–2020) were intentionally excluded to ensure temporal independence from the development cohort. Among them, 108 patients with tumor size < 4 cm comprised the temporally independent validation cohort (Fig. 1b). A prespecified subgroup analysis was performed in patients who were EGFR/ALK–negative or had unknown mutation status (*n* = 84).

### FDG-PET/CT assessment, cN staging, and pN evaluation

All patients were evaluated using integrated three-dimensional PET/CT scanners. cN staging was performed in accordance with NCCN [6], Japanese Lung Cancer Society [7], ESTS [9], and ACCP guidelines [10] at each institution.

In principle, mediastinal nodal evaluation by EBUS-TBNA and/or mediastinoscopy was performed in (i) patients with enlarged lymph nodes showing FDG uptake on PET/CT or contrast-enhanced CT or (ii) those with cN1 disease, centrally located tumors, or tumor size ≥ 3 cm, even in the absence of mediastinal FDG uptake whenever feasible [7, 9, 10]. By contrast, pathological mediastinal evaluation was considered optional for peripheral tumors located in the outer one-third of the lung with a diameter < 3 cm and no mediastinal FDG uptake on PET/CT [9, 10].

Although current guidelines do not uniformly define the indications for hilar EBUS-TBNA, FDG-avid hilar nodes were sampled whenever feasible. Otherwise, the decision was left to each institution’s discretion. If EBUS-TBNA was technically infeasible, cN staging was determined based on imaging findings through multidisciplinary discussion. Nodal positivity on PET/CT was qualitatively defined as focal FDG uptake greater than background activity [6, 7, 9, 10]. Tumor staging was performed in accordance with the 8th edition of the TNM classification for lung cancer [20].

Patients who underwent anatomical lung resection (segmentectomy, lobectomy, or pneumonectomy) with systematic or selective mediastinal lymph node dissection were included. Meanwhile, those who underwent lymph node sampling alone were excluded.

### Primary/secondary outcomes and definitions

The primary objective of this study was to identify predictive factors associated with cN(+)pN(−) disease and the secondary objective was to validate the corresponding prediction model. The representative imaging examples of cN(+)pN(−) cases are shown in Supplementary Fig. 1.

The site of recurrence was defined as the metastatic organ detected at the time of the first recurrence during follow-up. Intrathoracic recurrence included the hilar and mediastinal lymph nodes, lung, and pleura. Meanwhile, distant recurrence included the central nervous system, bone, and abdominal organs (e.g., liver and adrenal glands).

Recurrence-free survival (RFS) was defined as the interval from the date of surgery to recurrence or death from any cause. Patients without recurrence were censored at the last follow-up date. OS was defined as the interval from surgery to death from any cause. Patients who were alive at the last follow-up were censored at that time.

### Statistical analysis

Group comparisons were performed using the Mann–Whitney U test for continuous variables and Fisher’s exact test for categorical variables. Univariable logistic regression was used to assess associations between cN(+)pN(−) disease and age, sex, Brinkman index, tumor size, radiologic appearance (pure solid vs. part-solid), tumor laterality (right-sided), blood serum carcinoembryonic antigen (CEA) value, cN category (cN1 vs. cN2), tumor maximum standardized uptake value (SUVmax), and histology (squamous cell carcinoma). The cutoff values for age, Brinkman index, tumor size, serum CEA level, and SUVmax were determined using receiver operating characteristic (ROC) curve analysis, with cN(+)pN(−) disease as the endpoint. ROC-derived cutoff values were selected to maximize the prediction model’s discriminatory performance rather than to reflect predefined clinical thresholds. Variables with *p* < 0.10 in the univariable analyses were considered candidate predictors and subsequently entered into a multivariable logistic regression model. The independent predictors that were identified in the multivariable analysis were retained in the final prediction model. Statistical significance was considered at *p* < 0.05.

RFS and OS were estimated using the Kaplan–Meier method and compared using the log-rank test. Univariable and multivariable Cox proportional hazards regression analyses were performed to identify clinical factors that were independently associated with RFS and OS. Variables with *p* < 0.10 in the univariable analyses were entered into the multivariable Cox regression models.

Model discrimination was evaluated using the C-index. Calibration was assessed in the validation cohort (both overall validation cohort and in the EGFR/ALK-negative subset). To evaluate overfitting and internal validity, bootstrap internal validation with 1,000 resamples was performed in the development and validation cohorts, and optimism-corrected measures of discrimination and calibration were obtained.

Sensitivity analyses were performed using different thresholds for the number of predictive factors met (≥ 1, ≥ 2, ≥3, ≥ 4, and all 5). For each cutoff, the positive predictive value (PPV), specificity, and accuracy for cN(+)pN(−) disease were calculated in the development cohort, overall validation cohort, and EGFR/ALK-negative or unknown validation cohort.

All statistical analyses were performed using EZR (R Commander version 1.30; Saitama Medical Center, Jichi Medical University, Saitama, Japan), a graphical user interface for R (The R Foundation for Statistical Computing, Vienna, Austria).

## Results

### Development cohort

In the development cohort, 72 patients (28.7%) were classified as cN(+)pN(−), whereas 179 (71.3%) were classified as cN(+)pN(+). The baseline clinicopathological characteristics of patients are summarized in Table 1. The patients in the cN(+)pN(−) group were older (median, 73 vs. 70 years; *p* = 0.003), had higher frequency of right-sided tumors (76.4% vs. 55.9%; *p* = 0.003), were more often staged as cN1 (84.7% vs. 67.6%; *p* = 0.007), and had lower tumor PET SUVmax (median, 7.5 vs. 9.8; *p* = 0.039) compared with those in the cN(+)pN(+) group. No significant differences were observed between the two groups with regard to institution (*p* = 0.367) or operative period (*p* = 0.403). Among patients with cN(+)pN(−) disease, 61 patients (84.7%) were classified as cN1–pN0, and 11 patients (15.3%) were classified as cN2–pN0.

**Table 1:** Comparison of the characteristics between false-positive and non-false-positive patients with cN1–2 NSCLC

		cN(+)pN(+) (*n* = 179)	cN(+)pN(−) (*n* = 72)	*P*-value^a)^
Age, median (IQR), years		70 (64–76)	73 (69–77)	0.003^b)^
Male, No. (%)		134 (74.9)	48 (66.7)	0.212
Smoking history (+), No. (%)		142 (79.3)	57 (79.2)	1.000
Brinkman index, median (IQR)		800 (263–1155)	820 (170–1220)	0.869^b)^
Tumor size, median (IQR), cm		2.7 (2.3–3.3)	2.9 (2.3–3.3)	0.337^b)^
Consolidation tumor size, median (IQR), cm		2.7 (2.1–3.3)	2.7 (2.3–3.2)	0.486^b)^
Pure solid, No. (%)		153 (85.5)	61 (84.7)	0.847
Right side, No. (%)		100 (55.9)	55 (76.4)	0.003
CEA, median (IQR), ng/mL		5.6 (3.1–13.1)	4.9 (2.8–8.0)	0.050^b)^
PET SUVmax, median (IQR)		9.8 (6.2–14.1)	7.5 (4.3–14.1)	0.039^b)^
cN1, No. (%)		121 (67.6)	61 (84.7)	0.007
Operation period				
2010–2015		88 (49.2)	40 (55.6)	
2016–2020		91 (50.8)	32 (44.4)	0.403
Institution				
A		45 (25.1)	16 (22.2)	
B		79 (44.1)	39 (54.2)	
C		55 (30.7)	17 (23.6)	0.367
Squamous cell carcinoma, No. (%)		41 (22.9)	23 (31.9)	0.151
Extent of lung resection, No. (%)				
Segmentectomy		3 (1.7)	1 (1.4)	0.174
Lobectomy		168 (93.9)	71 (98.6)	
Pneumonectomy		8 (4.5)	0	
Lymphatic invasion, (%)		113 (63.1)	15 (20.8)	< 0.001
Vascular invasion, (%)		143 (79.9)	33 (45.8)	< 0.001
Pleural invasion, (%)		81 (45.3)	25 (34.7)	0.158
Patients with no known EGFR mutations or ALK rearrangements		150 (83.8)	56 (77.8)	0.278
Pathological stage, No. (%)				
p-stage IB or less		0	58 (80.6)	
p-stage II		70 (39.1)	14 (19.4)	
p-stage IIIA or more		109 (60.9)	0	
Lymph node status migration, No. (%)				
Down	cN1 pN0		61 (84.7)	
	cN2 pN0		11 (15.3)	
	cN2 pN1	4 (2.2)		
Up	cN1 pN2	45 (25.1)		
None	cN1 pN1	76 (42.5)		
	cN2 pN2	54 (30.2)		

^a)^ Fisher’s exact test

^b)^ Mann–Whitney U test

*ALK* anaplastic lymphoma kinase, *cN* clinical nodal status, *IQR* interquartile range, *EGFR* epidermal growth factor receptor, *NSCLC* non-small cell lung cancer, *PET* positron emission tomography, *pN* pathological nodal status, *SUVmax* maximum standardized uptake value

ROC curve analyses were used to determine cutoff values for continuous variables associated with cN(+)pN(−) (Supplementary Fig. 2). The optimal cutoffs were identified for age (67 years), tumor size (3.0 cm), Brinkman index (1000), tumor PET SUVmax (6.5), and serum CEA (12 ng/mL).

On multivariable logistic regression analysis, age ≥ 67 years (odds ratio [OR], 2.85; 95% confidence interval [CI], 1.40–5.79; *p* = 0.004), right-sided tumor (OR, 2.50; 95% CI, 1.30–4.82; *p* = 0.006), CEA ≤ 12 ng/mL (OR, 3.11; 95% CI, 1.01–9.53; *p* = 0.048), cN1 category (OR, 2.37; 95% CI, 1.12–5.04; *p* = 0.025), and tumor PET SUVmax ≤ 6.5 (OR, 2.03; 95% CI, 1.10–3.73; *p* = 0.023) were independently associated with cN(+)pN(−) disease (Table 2).

**Table 2:** Univariable and multivariable analyses of preoperative factors associated with cN(+)pN(−)

Variable	cN(+)pN(−)					
	Univariable analysis			Multivariable analysis		
	OR	95% CI	*P*-value	OR	95% CI	*P*-value
Age ≥ 67 years	2.91	1.49–5.70	0.002	2.85	1.40–5.79	0.004
Male	0.67	0.37–1.22	0.190			
Brinkman index ≥ 1000	1.45	0.83–2.55	0.193			
Tumor size ≥ 3.0 cm	1.39	0.80–2.42	0.239			
Pure solid	0.94	0.44–2.02	0.879			
Right side	2.56	1.38–4.75	0.003	2.50	1.30–4.82	0.006
CEA ≤ 12 ng/mL	4.28	1.46–12.5	0.008	3.11	1.01–9.53	0.048
cN1	2.66	1.30–5.43	0.007	2.37	1.12–5.04	0.025
Tumor PET SUVmax ≤ 6.5	2.44	1.38–4.31	0.002	2.03	1.10–3.73	0.023
Squamous cell carcinoma	1.58	0.86–2.90	0.139			

*CEA* carcinoembryonic antigen, *CI* confidence interval, *cN* clinical nodal status, *OR* odds ratio, *PET* positron emission tomography, *pN* pathological nodal status, *SUVmax* maximum standardized uptake value

**Fig. 2:**
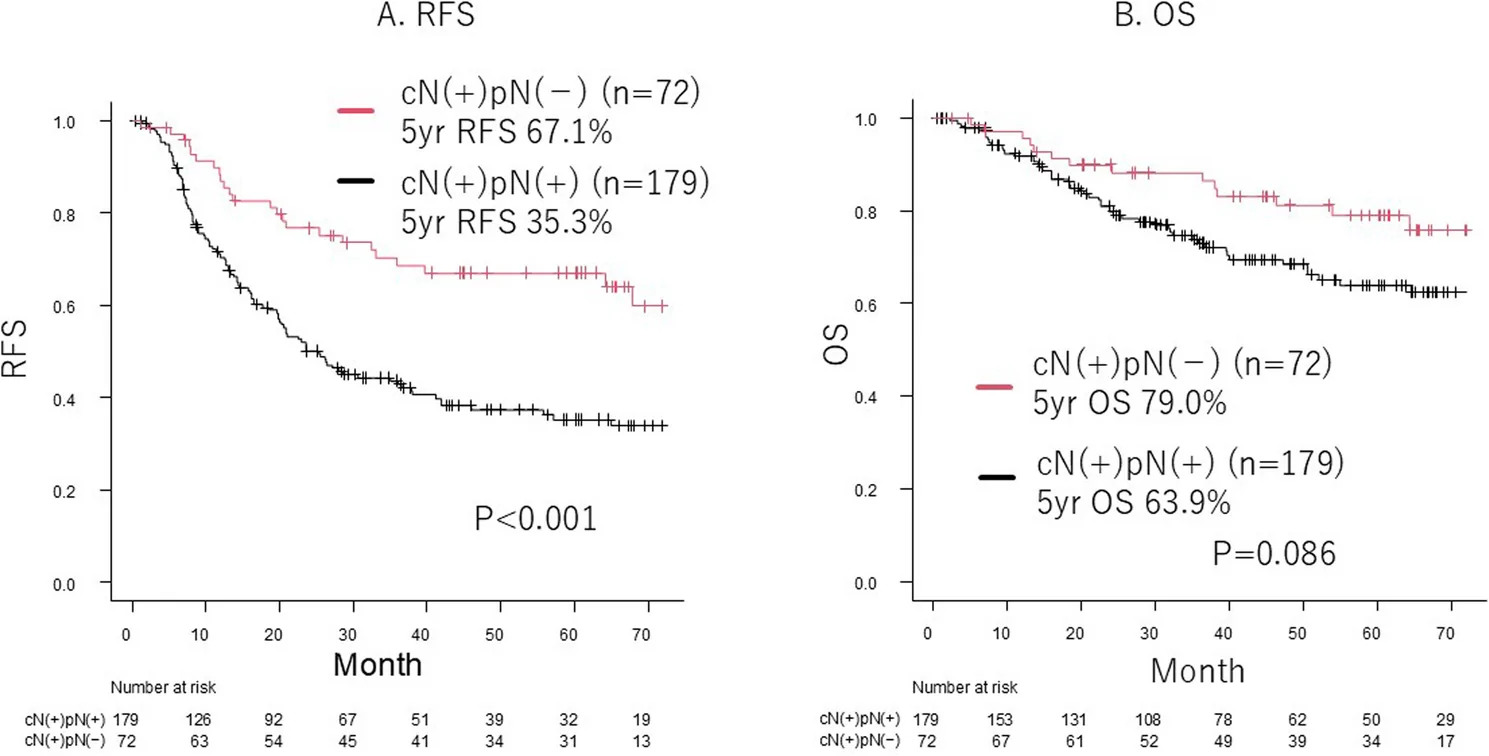
Survival outcomes according to pathological nodal status in clinically node-positive non-small cell lung cancer (< 4 cm). **A** Recurrence-free survival (RFS) and **B** overall survival (OS) in the development cohort stratified by pathological nodal status. Patients with cN(+)pN(−) disease (*n* = 72) demonstrated significantly better RFS than those with cN(+)pN(+) disease (*n* = 179) (5-year RFS: 67.1% vs. 35.3%, *p* < 0.001). The OS also tended to be better in the cN(+)pN(−) group than in the cN(+)pN(+) group (5-year OS: 79.0% vs. 63.9%, *p* = 0.086)

The cN(+)pN(−) group had significantly better RFS compared with the cN(+)pN(+) group (5-year RFS: 67.1% vs. 35.3%; *p* < 0.001). Moreover, the OS tended to be better in the cN(+)pN(−) group than in the cN(+)pN(+) group, although the difference did not reach statistical significance (5-year OS: 79.0% vs. 63.9%; *p* = 0.086) (Fig. 2). Multivariable Cox proportional hazards analyses adjusting for preoperative clinical variables confirmed that cN(+)pN(−) status remained independently associated with improved RFS (hazard ratio [HR], 0.51; 95% CI, 0.33–0.81; *p* = 0.004), whereas no independent association with OS was observed (HR, 0.65; 95% CI, 0.36–1.20; *p* = 0.169) (Supplementary Tables 1 and 2).

**Fig. 3:**
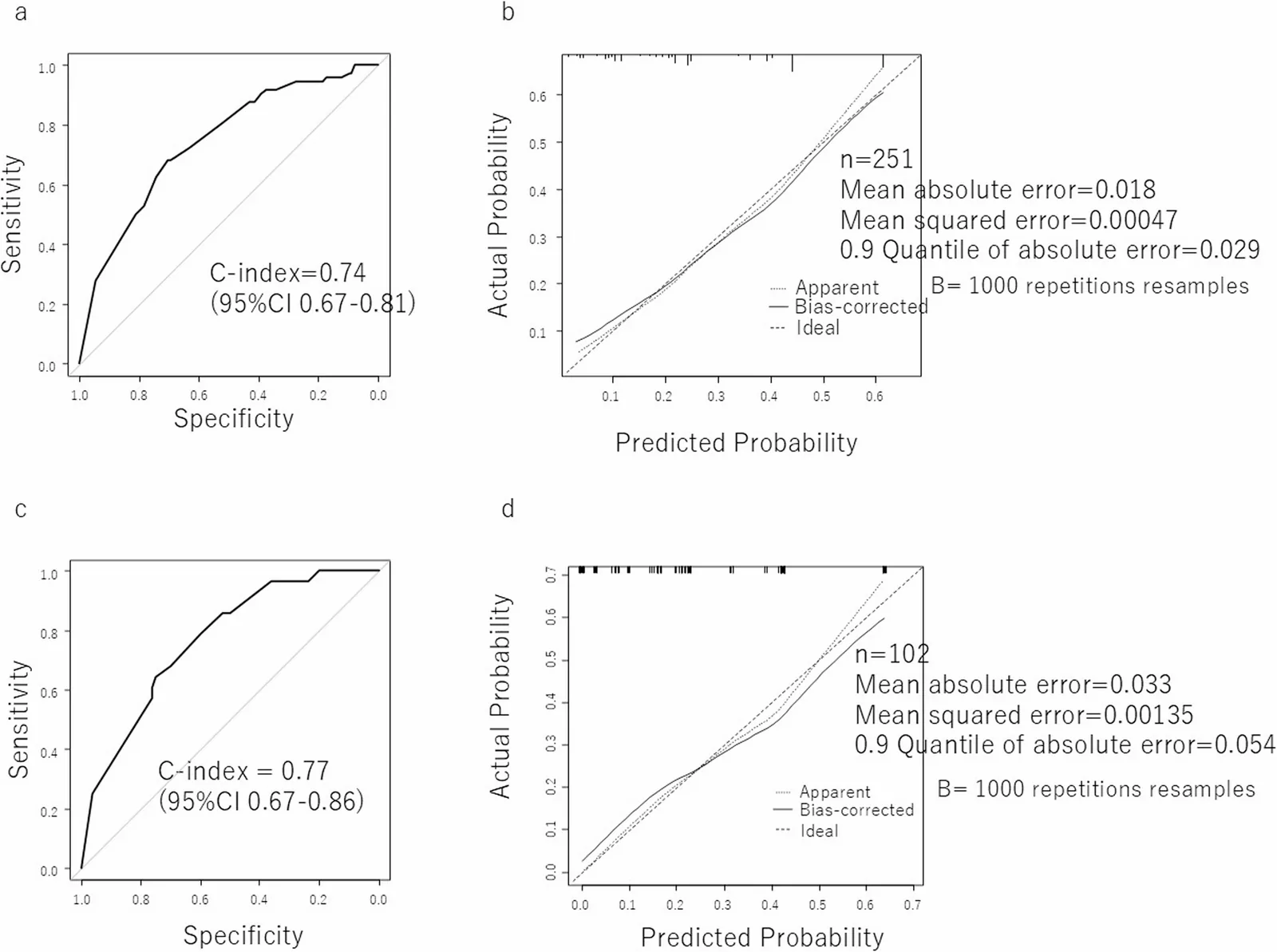
Prediction model’s performance in the development and validation cohorts. (a) Receiver operating characteristic (ROC) curve of the five-factor model for predicting cN(+)pN(−) disease in the development cohort (n = 251). The model demonstrated good discrimination, with a C-index of 0.74 (95% confidence interval: 0.67–0.81). (b) Calibration plot of the prediction model after internal validation using bootstrap resampling (1,000 repetitions), showing good agreement between predicted and observed probabilities. (c) Receiver operating characteristic (ROC) curve of the five-factor model in the validation cohort, demonstrating good discriminative performance (C-index = 0.77; 95% confidence interval, 0.67–0.86). (d) Calibration plot of the prediction model in the validation cohort after bootstrap resampling (1,000 repetitions), showing good agreement between predicted and observed probabilities

ROC curve analysis of the model incorporating the five predictive factors yielded a C-index of 0.74 (95% CI, 0.67–0.81) (Fig. 3a). The calibration showed good agreement between the predicted and observed probabilities (Fig. 3b). Among the 251 patients, 30 patients (12.0%) met all five predictive factors (age ≥ 67 years, right-sided tumor, CEA ≤ 12 ng/mL, cN1, and tumor SUVmax ≤ 6.5), of whom 20 (66.7%) were pN0. The PPV, specificity, and accuracy progressively increased as the number of predictive factors increased in the development cohort (Table 3). When all five predictive factors were present, the PPV was 66.7% (95% CI, 47.2%–82.7%), the specificity was 94.4% (95% CI, 90.0%–97.3%), and the accuracy was 75.3% (95% CI, 69.5%–80.5%).

**Table 3:** Performance of the prediction model for detecting cN(+)pN(−) disease according to the number of predictive factors in the development and validation cohorts

	Number of predictive factors	Positive predictive value (%)	Specificity (%)	Accuracy (%)
Development cohort, *n* = 251	≥ 1	29.1 (23.6–35.2)	2.2 (0.6–5.6)	30.3 (24.7–36.4)
	≥ 2	30.0 (24.2–36.4)	8.9 (5.2–14.1)	34.3 (28.4–40.5)
	≥ 3	35.9 (28.9–43.3)	34.1 (27.2–41.5)	50.6 (44.2–56.9)
	≥ 4	47.1 (37.2–57.2)	69.3 (62.0–75.9)	68.9 (62.8–74.6)
	5	66.7 (47.2–82.7)	94.4 (90.0–97.3)	75.3 (69.5–80.5)
Validation cohort (overall), *n* = 108	≥ 1	26.2 (18.1–35.6)	1.3 (0–6.8)	26.9 (18.8–36.2)
	≥ 2	27.7 (19.3–37.5)	8.8 (3.6–17.2)	32.4 (23.7–42.1)
	≥ 3	31.8 (22.1–42.8)	27.5 (18.1–38.6)	45.4 (35.8–55.2)
	≥ 4	43.2 (28.3–59.0)	68.8 (57.4–78.7)	68.5 (58.9–77.1)
	5	70.0 (34.8–93.3)	96.3 (89.4–99.2)	77.8 (68.8–85.2)
Validation cohort (EGFR/ALK-negative or unknown subset), *n* = 84	≥ 1	31.0 (21.3–42.0)	0 (0–9.1)	31.0 (21.3–42.0)
	≥ 2	32.5 (22.4–43.9)	6.9 (1.9–16.7)	35.7 (25.6–46.9)
	≥ 3	36.8 (25.4–49.3)	25.9 (15.3–39.0)	47.6 (36.6–58.8)
	≥ 4	47.2 (30.4–64.5)	67.2 (53.7–79.0)	66.7 (55.5–76.6)
	5	66.7 (29.9–92.5)	94.8 (85.6–98.9)	72.6 (61.8–82.9)

*ALK* anaplastic lymphoma kinase, *cN* clinical nodal status, *EGFR* epidermal growth factor receptor, *pN* pathological nodal status

### Validation in the overall validation cohort

In the validation cohort (*n* = 108), 28 patients (25.9%) were classified as cN(+)pN(−). The baseline clinicopathological characteristics of patients are summarized in Table 4. Similar trends were observed in the validation cohort: patients with cN(+)pN(−) disease were older, had higher frequency of right-sided tumors, more often had CEA ≤ 12 ng/mL and cN1 disease, and tended to have lower tumor SUVmax (Table 4).

**Table 4:** Comparison of the characteristics between false-positive and non-false-positive patients with NSCLC in the validation cohort

Total *n* = 108		cN(+)pN(+) (*n* = 80)	cN(+)pN(−) (*n* = 28)	*P*-value^a)^
Age, median (IQR), years		71 (65–76)	76 (71–77)	0.037^b)^
Male, No. (%)		47 (58.8)	20 (71.4)	0.266
Smoking history (+), No. (%)		61 (76.2)	21 (75.0)	1.000
Brinkman index, median (IQR)		633 (0–1208)	660 (158–840)	0.671^b)^
Tumor size, median (IQR), cm		2.8 (2.2–3.4)	2.7 (2.2–3.2)	0.384^b)^
Pure solid, No. (%)		77 (96.2)	21 (75.0)	0.003
Right side, No. (%)		46 (57.5)	23 (82.1)	0.023
CEA ≤ 12 ng/mL, No. (%)		69 (86.2)	28 (100)	0.063
PET SUVmax, median (IQR)		10.3 (7.3–13.9)	9.3 (3.3–12.2)	0.084^b)^
cN1, No. (%)		52 (65.0)	24 (85.7)	0.054
Squamous cell carcinoma, No. (%)		20 (25.0)	13 (46.4)	0.055
All five predictive factors		3 (3.8)	7 (25.0)	0.003
Lymphatic invasion, (%)		42 (52.5)	2 (7.1)	< 0.001
Vascular invasion, (%)		60 (75.0)	10 (35.7)	< 0.001
Pleural invasion, (%)		31 (38.8)	6 (21.4)	0.111
Pathological stage, No. (%)				
p-stage IB or less		0	23 (82.1)	
p-stage II		36 (45.0)	4 (14.3)	
p-stage IIIA or more		44 (55.0)	1 (3.6)	
Lymph node status migration, No. (%)				
Down	cN1 pN0		24 (85.7)	
	cN2 pN0		4 (14.3)	
	cN2 pN1	4 (5.0)		
Up	cN1 pN2	19 (23.8)		
None	cN1 pN1	33 (41.3)		
	cN2 pN2	24 (30.0)		

^a)^ Fisher’s exact test

^b)^ Mann–Whitney U test

*CEA* carcinoembryonic antigen, *cN* clinical nodal status, *IQR* interquartile range, *NSCLC* non-small cell lung cancer, *PET* positron emission tomography, *pN* pathological nodal status, *SUVmax* maximum standardized uptake value

ROC curve analysis demonstrated a C-index of 0.77 (95% CI, 0.67–0.86) (Fig. 3c). The calibration showed good agreement between the predicted and observed probabilities (slope, 1.002; intercept, 0.001) (Fig. 3d). Ten patients (9.3%) fulfilled all five predictive factors, of whom seven were pN0. The PPV, specificity, and accuracy progressively increased as the number of predictive factors increased in the overall validation cohort (Table 3). When all five predictive factors were present, the PPV was 70.0% (95% CI, 34.8%–93.3%), the specificity was 96.3% (95% CI, 89.4%–99.2%), and the accuracy was 77.8% (95% CI, 68.8%–85.2%).

### Validation in the EGFR/ALK-negative or unknown subset

**Fig. 4:**
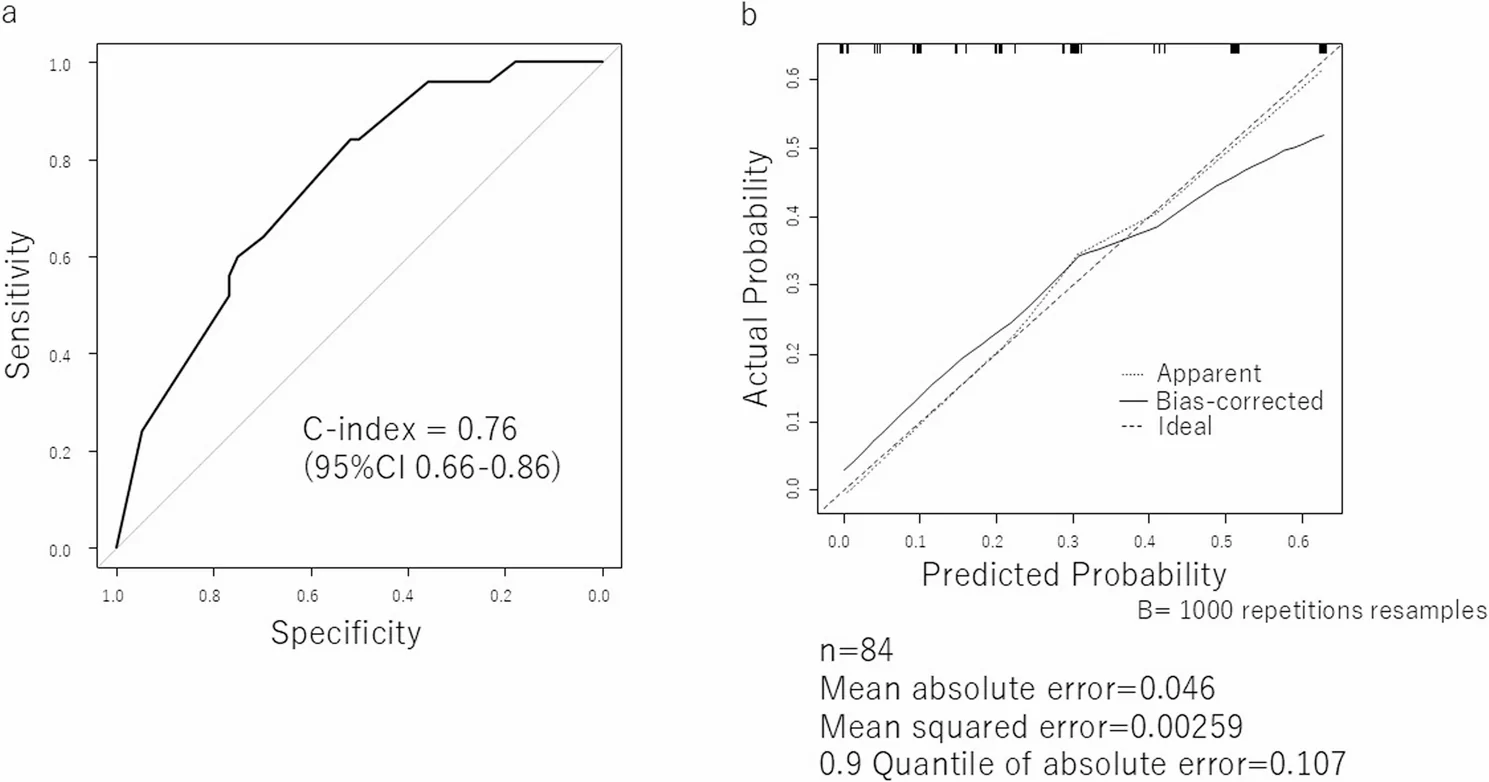
Validation of the prediction model in the epidermal growth factor receptor (EGFR)/anaplastic lymphoma kinase (ALK)–negative or unknown subset (*n* = 84). **a** Receiver operating characteristic (ROC) curve of the prediction model for cN(+)pN(−) disease in the EGFR/ALK-negative validation cohort, showing a C-index of 0.76 (95% confidence interval [CI], 0.66–0.86). **b** Calibration plot of the model in the same cohort, demonstrating good agreement between predicted and observed probabilities

Among patients without known EGFR mutations or ALK fusions in the validation cohort (*n* = 84), 26 patients (30.9%) were classified as cN(+)pN(−). The model achieved a C-index of 0.76 (95% CI, 0.66–0.86) (Fig. 4a) and demonstrated excellent calibration (slope, 1.001; intercept, 0.001) (Fig. 4b). Nine patients (10.7%) met all five predictive factors, of whom six were pN0. The PPV, specificity, and accuracy progressively increased as the number of predictive factors increased in the EGFR/ALK-negative or unknown validation cohort (Table 3). When all five predictive factors were present, the PPV was 66.7% (95% CI, 29.9%–92.5%), the specificity was 94.8% (95% CI, 85.6%–98.9%), and the accuracy was 72.6% (95% CI, 61.8%–82.9%).

## Discussion

In this multicenter study of < 4-cm cN(+) NSCLC, 28.7% of patients in the development cohort were classified as cN(+)pN(−), indicating a substantial rate of false-positive cN staging. These patients demonstrated significantly better RFS than those with cN(+)pN(+) disease. Five independent preoperative factors associated with cN(+)pN(−) disease were identified, including age ≥ 67 years, right-sided tumor, CEA ≤ 12 ng/mL, cN1, and tumor SUVmax ≤ 6.5. The resulting model achieved PPVs of 70.0% and 66.7% and high specificity (96.3% and 94.8%) in the overall validation cohort and the EGFR/ALK-negative or unknown subset, suggesting that it can identify patients likely to be cN(+)pN(−) while minimizing misclassification of true pN(+) disease.

Several randomized trials have established the benefit of neoadjuvant chemo-immunotherapy and perioperative immunotherapy in resectable NSCLC [3–5, 19]. According to subgroup analyses from the CheckMate 816, KEYNOTE-671, and AEGEAN trials, the treatment effect was more pronounced in patients with more advanced stage disease, particularly those with stage III or nodal involvement (e.g., HR 0.54 in stage III vs. 0.87 in stage IB–II in the CheckMate 816 trial; HR 0.57 in stage IIIA vs. 0.76 in stage II in the AEGEAN trial) [3–5]. Moreover, in the CheckMate 816 trial, patients with higher tumor mutational burden (≥ 12.3 mut/Mb) derived greater benefit, further supporting the concept that preoperative immune checkpoint inhibition is particularly advantageous in patients with true nodal metastasis and higher systemic disease burden [3]. These findings support the biological rationale that neoadjuvant immune checkpoint inhibition is particularly beneficial in patients with true nodal metastasis and a higher burden of micrometastatic disease. In these patients, early systemic therapy may eradicate occult distant disease and improve long-term outcomes. By contrast, patients with cN(+)pN(−) disease lack pathological nodal involvement and likely have a lower systemic disease burden, potentially deriving limited benefit from preoperative systemic therapy while remaining exposed to its toxicities, ultimately resulting in overtreatment [3–5, 19].

Most previous studies that evaluated the accuracy of preoperative nodal staging included heterogeneous populations across tumor sizes and disease stages. Conversely, the present study specifically focused on tumors < 4 cm with cN(+) disease, a clinically important subgroup that is a candidate for neoadjuvant chemo-immunotherapy. Despite evaluation at three high-volume institutions, cN staging remained suboptimal, underscoring the need for improved strategies to refine preoperative nodal assessment.

The accuracy of cN staging based on CT and PET/CT remains limited, particularly because of suboptimal specificity and PPV reported across studies [21–26]. Therefore, guidelines recommend pathological confirmation using EBUS/EUS with mediastinoscopy in selected cases [6, 7, 9, 10, 27]. Although EBUS-TBNA provides high diagnostic accuracy for accessible stations [13], accurate staging remains challenging for anatomically difficult stations, including several N1 nodes (e.g., #10, #12–14) [11, 12, 28]. Consistent with these limitations, most false-positive cases in the present study were cN1 (84.7% in the development cohort and 85.7% in the validation cohort), leaving clinicians to primarily rely on imaging when deciding on neoadjuvant therapy.

False positivity of cN staging is a well-recognized limitation of PET/CT-based nodal assessment. Previous studies have reported false-positive rates of approximately 20%–50% for hilar and mediastinal lymph nodes when PET/CT is used alone [29, 30]. Lower nodal SUVmax, older age, and non-adenocarcinoma histology have been associated with false-positive findings [29, 31], and inflammatory or granulomatous conditions may further contribute to increased FDG uptake and false-positive interpretations [30, 32–34]. These observations are consistent with our finding that false-positive cN staging occurred more frequently among older patients.

Inaccurate preoperative nodal staging has been consistently reported. A meta-analysis showed low concordance between cTNM and pTNM staging, with overstaging in approximately 14% of cases [14]. Solberg et al. reported a cTNM–pTNM concordance of 48.1%, with cN(+)pN(–) disease being observed in 22.8% of patients [17]. Meanwhile, Macia et al. found an overall concordance of only 58%, which was particularly low in stages IB–III [18]. Together with the present findings, these data highlight the inherent limitations of preoperative nodal staging and the clinical importance of accounting for false-positive cN disease.

The prediction model identified in this study, based on five readily available preoperative factors, enables high-specificity identification of patients with < 4-cm cN(+) NSCLC who are unlikely to have pathological nodal metastasis. This is particularly relevant for patients with cN1 disease, in whom pathological confirmation by EBUS/EUS may be technically challenging or incomplete because not all hilar lymph node stations are readily accessible. The proposed model is intended to complement, rather than replace, standard pathological nodal evaluation whenever feasible, including EBUS/EUS-guided sampling and mediastinoscopy. Accordingly, it may serve as a supportive risk-stratification tool for clinical decision-making in situations where pathological confirmation is not feasible or remains inconclusive. The model may help identify patients at high risk of cN(+)pN(−) disease in whom upfront surgery for definitive pathological staging may be considered after comprehensive clinical assessment, thereby helping to avoid unnecessary neoadjuvant immunotherapy while preserving appropriate treatment for patients with true pathological nodal metastasis.

This study has several limitations. First, its retrospective design may have introduced selection bias and bias in treatment decision-making, which cannot be fully eliminated. Second, FDG-PET/CT scanners, imaging protocols, and methods for measuring SUVmax were not fully standardized across institutions, potentially limiting the reproducibility and generalizability of SUVmax measurements. Therefore, the model’s predictive performance may vary across institutions using different PET/CT systems or imaging protocols. Third, although preoperative nodal staging generally followed contemporary clinical guidelines, the diagnostic work-up was not completely standardized across institutions or patients because of the study’s retrospective design. Furthermore, the indications for and technical performance of EBUS/EUS and mediastinoscopy may have varied among institutions and operators, which could have influenced cN staging accuracy. Fourth, several ROC-derived cutoff values that were included in the prediction model may not correspond to commonly used clinical thresholds because they were statistically selected to maximize the prediction model’s discriminatory performance. Finally, temporal heterogeneity between the development and validation cohorts may have influenced the model’s performance because imaging technologies, including CT and FDG-PET/CT, as well as EBUS availability and clinical staging practices, have evolved over time. Although the model was developed using a multicenter cohort, validation was performed using a temporally independent cohort from a single institution, rather than an independent external cohort. Therefore, further external validation in independent cohorts reflecting contemporary clinical practice is warranted.

## Conclusion

Among patients with < 4-cm cN(+) NSCLC, approximately 30% had false-positive cN staging. These patients demonstrated significantly better RFS than those with pathological nodal metastasis. The prediction model based on the five preoperative factors identified in this study enabled high-specificity identification of cN(+)pN(–) cases, and its validity was confirmed in a temporally independent validation cohort. In the contemporary treatment landscape where neoadjuvant immunotherapy-based regimens are increasingly being adopted, this model may serve as a supportive risk-stratification tool to assist multidisciplinary clinical decision-making regarding upfront surgery, in conjunction with standard pathological nodal evaluation whenever feasible. 

## Supplementary Information

Supplementary Material 1: Supplementary Figure 1. Positron emission tomography/computed tomography (PET/CT) demonstrated high fluorodeoxyglucose (FDG) accumulation in hilar lymph node station #11s, leading to a clinical diagnosis of nodal metastasis. However, pathological examination of the dissected lymph nodes revealed no malignant cells, resulting in a final classification of cN(+)pN(−).

Supplementary Material 2: Supplementary Figure 2. Receiver operating characteristic (ROC) analyses for determining optimal cutoff values for continuous variables associated with cN(+)pN(−) disease.

Supplementary Material 3: Supplementary Table 1. Univariable and multivariable analyses for RFS.

Supplementary Material 4: Supplementary Table 2. Univariable and multivariable analyses for OS.

## Data Availability

The datasets used and/or analyzed during the current study are available from the corresponding author on reasonable request.

## Abbreviations

ACCP: American College of Chest Physicians
ALK: Anaplastic lymphoma kinase
CEA: Carcinoembryonic antigen
CI: Confidence interval
cN: clinical nodal status
CT: Computed tomography
cTNM–pTNM: clinical TNM–pathologic TNM
EBUS: Endobronchial ultrasound
EBUS-TBNA: Endobronchial ultrasound-guided transbronchial needle aspiration
EGFR: Epidermal growth factor receptor
EUS: Endoscopic ultrasound
ESTS: European Society of Thoracic Surgeons
FDG: Fluorodeoxyglucose
NCCN: National Comprehensive Cancer Network
NSCLC: Non-small cell lung cancer
OR: Odds ratio
OS: Overall survival
pN: pathological nodal status
PET/CT: Positron emission tomography/computed tomography
PPV: Positive predictive value
RFS: Recurrence-free survival
ROC: Receiver operating characteristic
SUVmax: Maximum standardized uptake value
TNM: Tumor, node, metastasis
