# Supplementary material for: A multicenter prediction model for false-positive clinical nodal disease in < 4-cm NSCLC: clinical implications for upfront surgery versus neoadjuvant therapy
Source: BMC Cancer. 2026 Jul 17;26:1221. doi: 10.1186/s12885-026-16580-4 (PMC13660139; doi:10.1186/s12885-026-16580-4)
Supplement: Supplementary file 1 [file 12885_2026_16580_MOESM3_ESM.docx]

Supplementary Table 1. Univariable and multivariable analyses for RFS

| Variable | Univariable analysis | | | Multivariable analysis | | |
| --- | --- | --- | --- | --- | --- | --- |
|  | HR | 95% CI | P-value | HR | 95% CI | P-value |
| cN(+)pN(−) status | 0.43 | 0.28–0.66 | <0.001 | 0.51 | 0.33–0.81 | 0.004 |
| Age ≥67 years | 1.03 | 0.72–1.47 | 0.894 |  |  |  |
| Male | 1.25 | 0.84–1.87 | 0.277 |  |  |  |
| Brinkman index ≥1000 | 1.22 | 0.86–1.74 | 0.257 |  |  |  |
| Tumor size ≥3.0 cm | 1.33 | 0.94–1.87 | 0.105 |  |  |  |
| Pure solid | 1.01 | 0.62–1.64 | 0.984 |  |  |  |
| Right side | 0.71 | 0.50–0.99 | 0.048 | 0.79 | 0.55–1.12 | 0.185 |
| CEA ≤12 ng/mL | 0.68 | 0.44–1.05 | 0.083 | 0.83 | 0.53–1.29 | 0.411 |
| cN1 | 0.40 | 0.28–0.58 | <0.001 | 0.44 | 0.31–0.63 | <0.001 |
| Tumor PET SUVmax ≤6.5 | 0.725 | 0.50–1.06 | 0.095 | 0.91 | 0.62–1.35 | 0.646 |
| Squamous cell carcinoma | 0.89 | 0.59–1.33 | 0.556 |  |  |  |

CEA, carcinoembryonic antigen; CI, confidence interval; cN, clinical nodal status; HR, hazard ratio; PET, positron emission tomography; pN, pathological nodal status; RFS, recurrence-free survival; SUVmax, maximum standardized uptake value
