# Supplementary material for: A multicenter prediction model for false-positive clinical nodal disease in < 4-cm NSCLC: clinical implications for upfront surgery versus neoadjuvant therapy
Source: BMC Cancer. 2026 Jul 17;26:1221. doi: 10.1186/s12885-026-16580-4 (PMC13660139; doi:10.1186/s12885-026-16580-4)
Supplement: Supplementary file 2 [file 12885_2026_16580_MOESM4_ESM.docx]

Supplementary Table 2. Univariable and multivariable analyses for OS

| Variable | Univariable analysis | | | Multivariable analysis | | |
| --- | --- | --- | --- | --- | --- | --- |
|  | HR | 95% CI | P-value | HR | 95% CI | P-value |
| cN(+)pN(−) status | 0.61 | 0.35–1.08 | 0.089 | 0.65 | 0.36–1.20 | 0.169 |
| Age ≥67 years | 2.58 | 1.41–4.73 | 0.002 | 2.81 | 1.52–5.21 | 0.001 |
| Male | 2.40 | 1.23–4.70 | 0.010 | 1.91 | 0.93–3.94 | 0.080 |
| Brinkman index ≥1000 | 1.95 | 1.22–3.13 | 0.006 | 1.72 | 1.02–2.90 | 0.041 |
| Tumor size ≥3.0 cm | 1.64 | 1.02–2.62 | 0.041 | 1.67 | 1.03–2.72 | 0.039 |
| Pure solid | 0.81 | 0.44–1.51 | 0.511 |  |  |  |
| Right side | 0.56 | 0.35–0.90 | 0.017 | 0.78 | 0.47–1.27 | 0.316 |
| CEA ≤12 ng/mL | 0.61 | 0.35–1.09 | 0.096 | 0.54 | 0.30–1.00 | 0.050 |
| cN1 | 0.43 | 0.26–0.69 | <0.001 | 0.44 | 0.26–0.72 | 0.001 |
| Tumor PET SUVmax ≤6.5 | 0.64 | 0.37–1.1 | 0.107 |  |  |  |
| Squamous cell carcinoma | 1.67 | 1.01–2.78 | 0.047 | 1.63 | 0.96-2.79 | 0.072 |

CEA, carcinoembryonic antigen; CI, confidence interval; cN, clinical nodal status; HR, hazard ratio; OS, overall survival; PET, positron emission tomography; pN, pathological nodal status; SUVmax, maximum standardized uptake value
